# Fall-Risk-Increasing Drugs in People With Dementia Who Live in a Residential Aged Care Facility: A Pilot Study

**DOI:** 10.7759/cureus.24559

**Published:** 2022-04-28

**Authors:** Caroline M Harris, Tatiana Lykina

**Affiliations:** 1 Medical School, Oceania University of Medicine, Corryong, AUS; 2 Allergy and Immunology, Oceania University of Medicine, Saint Petersburg, RUS

**Keywords:** serotonin, fall injury, depression in dementia, behavioural and psychological symptoms of dementia (bpsd), psychoactive drugs

## Abstract

Background

Psychotropic medications feature in prescribing guidelines for the treatment of depression in dementia as well as the management of *behavioural and psychological symptoms of dementia *(BPSD). They include antidepressants, antipsychotics, and benzodiazepines, and are among an established collective of pharmacotherapies known as fall-risk-increasing drugs (FRIDs). These psychoactive medications are known to increase fall risk in elderly adults, including those with a dementia diagnosis. Medication reviews are an integral part of falls prevention programs in residential aged care and provide an opportunity to modify medications to reduce fall risk related to pharmacotherapy.

Objectives

This pilot study explores the characteristics of a group of elderly people with dementia living in residential care with a focus on patterns of falls and usage of psychotropic medications.

Methods

This is a retrospective study conducted using data collected from health records. The Neuroscience-based Nomenclature (NbN) classification of psychotropic medicines is employed to highlight relevant pharmacological domains targeted by the medications rather than traditional drug classes.

Results

Four pharmacological neurotransmitter domains emerged as key players in the pharmacotherapy of study participants. These were serotonin, dopamine, noradrenaline, and gamma-aminobutyric acid A (GABA-A). Serotonin was the most frequently implicated domain as related to observed usage of psychotropic treatments for depression and BPSD. Over the retrospective study period, 75% of participants were taking prescribed psychotropics known to target these four domains, and most (69.4%) were elderly women over the age of 80. Many participants experienced multiple falls, mostly among women, and most falls were rated as harmful to some degree.

Conclusion

This study observes recurrent falls and frequent usage of psychoactive drugs in elderly people with dementia. We conclude that further investigations are both warranted to support prescribing guidelines for dementia and feasible according to the methodology of this pilot study.

## Introduction

Falls among the elderly are recognised as an increasing global health burden and a significant cause of morbidity and mortality [1]. Studies have confirmed the role of several classes of medications in increasing falls risk in older adults [2-4]. Although an international consensus list of fall-risk-increasing drugs (FRIDs) has yet to be created [5], current players include loop diuretics, psychotropic medications, and opioids. Classification of 'psychotropics' (psychotropic medications) has traditionally included antipsychotics, antidepressants, benzodiazepines, and mood stabilisers, and is a broad term used by many authors [3]. Efforts to clarify the nomenclature of psychotropics have emerged in recent years [6]. Psychotropic medications commonly studied in relation to falls risk are antipsychotics, antidepressants, and benzodiazepines (Appendix 1). Polypharmacy is also reported as being associated with an increased risk of falls in older adults [5,7].

In attempts to minimise falls related to prescribed pharmacotherapies, medication reviews feature as an integral part of falls prevention strategies in older adults [8], although the efficacy of this approach is unclear [9,10]. Importantly, the prevention of falls is a multidisciplinary area of care employing a wide range of strategies, including interventions not related to medications [8].

People with dementia living in residential care form an interesting subgroup of the elderly regarding falls because they are often prescribed medications known to be associated with an increased risk of falls [11-13]. Psychotropic medications are frequently singled out in the literature as key culprits for increased risk of falls in older adults [14]. These medicines feature in prescribing schedules for the management of people with dementia [15]. Due to concerns regarding the association of these psychoactive drugs with falls in older adults, prescribing trends in this area have come under scrutiny [14,16], although some studies report good practices [17].

Alzheimer’s disease is a common form of dementia, and therapeutic guidelines assist physicians in the choice of medications aimed at delaying the progression of this incurable degenerative condition and for the management of symptoms [15,18]. Non-pharmacological and multi-modal approaches are advised in the first instance [18], and current pharmacotherapies for managing cognitive impairment in dementia include cholinesterase inhibitors and memantine [19]. These drugs do not seem to be associated with an increased risk of falls [20].

The neurochemistry of agitation associated with Alzheimer’s is unclear and treatments for dementia are still being evaluated [19]. Regardless of the mechanisms underlying degenerative processes, behavioural disturbances and personality changes associated with dementia are known to be important risk factors for falls [8]. These behaviours and changes are known collectively as behavioural and psychological symptoms of dementia (BPSD) [21].

Clinicians may be prompted by guidelines to prescribe psychotropics for the management of BPSD. These symptoms include psychoses, aggression, nocturnal disruption, and wandering behaviours [21]. Published evidence, however, indicates that psychotropic medications are associated with an increased risk of falls, specifically in dementia groups [11-13]. Moreover, the withdrawal of psychotropics has been associated with a decreased risk of falls in the elderly, although not in dementia groups. [22]. A more recent systematic review, however, does not draw clear conclusions from the evidence base regarding recommended interventions for falls prevention in people with dementia, including the role of medications [23].

Psychotropic medications affect neurotransmitters in the brain in a variety of ways, although their mechanisms of action are only partially understood (Appendix 1). Serotonin, also known as 5-hydroxytryptamine, or 5-HT, is an important neurotransmitter and drug target in the brain. Serotonin enhancement (via reuptake inhibition) is mediated by antidepressant medicines such as selective serotonin reuptake inhibitors (SSRIs: escitalopram, citalopram, fluoxetine, sertraline, paroxetine) and serotonin and noradrenaline reuptake inhibitors (SNRIs: venlafaxine, duloxetine). Conversely, serotonin inhibition via blockade of central serotonin receptors is a mechanism of action of other antidepressants (mirtazapine), as well as the antipsychotics risperidone and quetiapine. Risperidone and quetiapine are both believed to act via the antagonism of central dopamine, serotonin, and noradrenaline receptors, and are therefore denoted as DSN antagonists in this report. The antipsychotic effects of both risperidone and quetiapine may be due to a combinatory antagonism at 5-HT2 receptors (higher selectivity) relative to D2 receptors. The noradrenergic blockade (central α1 adrenergic receptor antagonism) may contribute to orthostatic hypotension, often seen as an adverse effect of these medicines.

Haloperidol does not feature in dementia-specific protocols, although it is indicated for restless and impulsive behaviours in the elderly. Haloperidol is a typical (first generation) antipsychotic known to dampen dopaminergic pathways in the brain by competitive blockade of dopamine (D2) receptors.

Modulators binding gamma-aminobutyric acid A (GABA-A) receptors (benzodiazepines: oxazepam, temazepam, lorazepam, midazolam) are ligands that enhance inhibition of neurotransmission via chloride ion flux through GABA-A channels. Benzodiazepines, therefore, do not affect serotonergic, dopaminergic, or noradrenergic pathways.

Physicians seeking alternatives to antipsychotics for the management of BPSD may instead prescribe anticonvulsants [20]. Although anticonvulsants have previously been associated with an increased risk of falls in the elderly, according to earlier reviews [4], other studies find no significant association between anticonvulsants and increased fall risk in dementia [20]. Regarding the topic of FRIDs overall, reviewers observe a lack of studies investigating the possible effects of medication sub-groups and variable doses on fall risk in the elderly [2,3], as well as a lack of convincing evidence that might inform prescribing guidelines and interventions for supporting the care of people with dementia regarding medication-related falls [23]. A recent European Task and Finish Group suggests that there is a need for dissemination of knowledge and resources regarding FRIDs and falls across health care settings, as well as education for physicians to ensure safe prescribing and prudent withdrawal of pharmacotherapies in elderly populations [5].

Elderly females are known to have a higher rate of falls compared to men of the same age bracket, although the risk factors appear to be gender-based [24]. However, this phenomenon represents elderly populations in general and not dementia groups. The female gender is, however, a known risk factor for dementia [25].

This pilot study explores the patterns and characteristics of falls in the context of psychotropic medication usage in a small cohort of elderly people with dementia living in a residential care facility. We anticipate a pattern of increased falls in the context of psychotropic medication usage (as compared to non-usage) and expect a higher rate of falls among female participants. Overall, we are interested in any emergent themes that may assist in formulating hypotheses and informing feasibility for future studies regarding FRIDs and falls in the context of dementia.

## Materials and methods

Setting

Data were collected by retrospective chart abstraction from clinical records of residents with a diagnosis of dementia living in a residential aged care facility (RACF) in rural Australia. Ethics approval, including a consent waiver, was obtained prior to the commencement of data collection. The ethics committee defined a 5½-year study period based on the ethics approval date to ensure retrospective data collection. Data on falls and psychotropic medications administered over the study period were gathered and both electronic and paper-based records were consulted. The facility is not a designated dementia care unit but is a general aged care setting and has a 34-bed capacity with approximately a third of residents diagnosed with dementia at any given time (this figure fluctuates according to resident admissions, discharges, and deaths).

Falls data

A fall is defined by the World Health Organisation as 'an event which results in a person coming to rest inadvertently on the ground or floor or other lower level' [26]. A near miss (defined as an event where an individual almost has a fall but is rescued by self or others) is also included in the definition of a fall for the purposes of incident reporting. Near-miss incidents are as important as falls for shaping falls prevention strategies for individuals [8]. Falls and near-miss events are recorded by attending care staff via a formal incident reporting system and are allocated an incident severity rating (ISR) code according to the degree of harm sustained from the fall. Both falls incidence and ISR data were collected.

Data for bed occupancy were also collected to calculate the incidence rates of falls, which are usually reported by institutions as the number of falls per 1000 occupied bed days (OBD).

Data collection criteria

The data collection period extended from January 1, 2016 to June 30, 2021, a retrospective study period of 5½ years. Reasons for ending data collection were commencement of an end-of-life palliative care pathway, death, or discharge.

The study focuses on the following three psychotropic medication groups: benzodiazepines, antipsychotics, and antidepressants. The pharmacological classification of drugs according to the Neuroscience-based Nomenclature (NbN) 2nd edition revised is employed in this study [6]. The NbN is an innovative and updated classification system aimed at shifting from symptoms and disease to the pharmacological mechanisms of psychoactive medications. Examples of psychotropic medications prescribed for dementia clients in the study include risperidone, quetiapine, and haloperidol (formerly classified as antipsychotic); diazepam, oxazepam, lorazepam (benzodiazepines - formerly classified as anxiolytic or hypnotic), escitalopram, sertraline, and mirtazapine (formerly classified as antidepressants) (Appendix 1).

Medication charts were consulted to verify prescriptions and doses administered by care staff at the time of each fall incident. Doses, frequency, route, and class of medication were all recorded. Care was taken to ensure that prescribed medications were indeed administered. Many PRN medications are prescribed but given sparingly, if at all, and it is not uncommon for regular medicines to be declined by residents or withheld by clinicians.

The following four datasets were collected: (i) the participant is administered psychotropic medication and has falls; (ii) the participant is not administered psychotropic medication and has falls; (iii) the participant is administered psychotropic medication and has no falls; (iv) the participant is not administered psychotropic medication and has no falls.

These datasets are organized into two groups for the purposes of data analysis, namely fallers (participants who had at least one fall reported by attending care staff), and non-fallers (participants who had no falls reported during the study period).

Inclusion criteria

Participants were identified as clients who reside at the facility as either permanent or temporary residents. Temporary clients are denoted as 'respite clients'. Respite clients enjoy short-term stays at the facility, often several times over a period of years, staying for a week or several consecutive weeks at a time. Some of these respite clients had dementia diagnoses and resided at the facility during the study period. This allowed their inclusion in the study.

All participants had a medical diagnosis of dementia documented in their clinical history. Dementia is now regarded as an umbrella term for a group of conditions entitled 'major neurocognitive disorder' (major NCD) and includes Alzheimer’s disease, vascular dementia, Parkinson-related dementia, Lewy body dementia, and other forms of major NCD. All types of dementia were included in the study according to DSM-V diagnostic criteria for major neurocognitive disorders [27].

Both ambulant and non-ambulant participants were included. Non-ambulant residents are at risk of falls from a bed, chair, or equipment used for transfers. Participants were included even if they had no fall events during the study period. Participants were included even if they were not being administered psychotropic medications during the study period. Each participant received a study ID number to allow de-identification of data (Appendices 3 and 4). The age of participants was designated either as their age on January 1, 2016 (the start of the study period) or their age at dementia diagnosis following admission into the facility, whichever was the latter.

Exclusion criteria

Any resident without a diagnosis of dementia was excluded from the study. The study explored resident-related factors in dementia clients only. There were no age or gender exclusion criteria in the study, as this research is purely a descriptive, exploratory pilot study. It is uncertain whether fall risk relates to age or gender in dementia groups [11], although it is known that in the general population, older females are more likely to fall than their male counterparts [25].

Due to a catastrophic bushfire in 2020 that passed through the rural region where the facility is located, a period of zero bed occupancy in the facility arose as the residents were all evacuated. The evacuation occurred over a period of four hours and incurred no falls. The residents remained absent for a period of approximately a month during January and February 2020. This effectively shortened the study period by a month but still allowed the calculation of fall incidence rates, which are derived from bed occupancy data.

Statistical analysis

Descriptive analysis was performed with counts, percentages, measures of dispersion, and odds ratios (OR). Tabular analysis and chi-square tests of proportion were conducted to obtain P-values. The P-values were p<0.05 for gender distribution in the dementia study cohort and p<0.01 for gender distribution among fallers.

## Results

The study period was defined according to ethics approval and extended from January 1, 2016 to June 30, 2021, covering 5½ years. This ensured the collection of retrospective data only.

During the study period, a total of 94 people were living in the facility, either on a permanent or temporary residential care basis. Three of the 94 clients were disability clients and not elderly residents. They were aged under 65 at the time of the first admission and lived at the facility permanently due to a need for full-time professional care for reasons other than advanced age or dementia. These three clients were excluded from the count, leaving a total of 91 residents. Of these 91 residents, 36 (39.5%) had a documented dementia diagnosis. These 36 residents were selected as participants, forming the study cohort (n=36).

Age and gender

Most participants were long-term residents of the local community, with a few arriving from outside the area to benefit from residential care close to local family members. Of the 36 residents in the study cohort, 25 (69.4%) were female and 11 (30.6%) were male, deemed statistically significant at p<0.05 (χ^2^ = 5.444, p = 0.01963). The mean age of participants was 83.50 years (SD=6.068), with a range from 70 to 94 years old. The mean female age was 83.68 years (SD=5.490) with a range from 70 to 94 years old. The mean male age was 83.09 years (SD=7.502) with a range from 73 to 94 years old.

Dementia diagnosis

Most participants (n=25, 69.4%) had a diagnosis of dementia at the time of the commencement of the study period. Those who were diagnosed with dementia during the study period (n=11; 30.6%) only had their data analysed from the date of their diagnosis.

The types of major NCD (dementia) diagnosed among the participants are shown in Table 1. Half of the major NCD diagnoses found in the medical records were simply entered as 'dementia' with no documentation of a specific dementia type (column 1, Table 1: the authors have denoted 'unspecified dementia' for these participants). Alzheimer's dementia was the most common of the specified diagnoses. Several cases of vascular dementia occurred, and a single case of major NCD related to Parkinson’s disease, alcoholism, and BPSD.

**Table 1:** Types of major NCD encountered in the study NCD: neurocognitive disorder, BPSD: behavioural and psychological symptoms of dementia

Major NCD types	Total (n=36)	%
Unspecified dementia	18	50.0%
Alzheimer disease	12	33.3%
Vascular dementia	3	8.3%
BPSD-related dementia	1	2.8%
Parkinson-related dementia	1	2.8%
Alcohol-related dementia	1	2.8%

Falls and medications

Table 2 summarises the data on falls and psychoactive medications. Participants in the faller group (n=28) had at least one fall during the study period. Participants of the non-faller group (n=8) had no falls during the study period. A total of 121 falls were reported over the study period for the entire cohort (n=36). Of the 36 dementia clients, 28 (77.8%) sustained these falls. The remaining eight dementia clients (22.2%) had no falls during the study period. Each group comprised two sub-groups as defined by the administration (or absence) of prescribed psychotropics. In both groups (fallers and non-fallers), participants receiving psychotropic medication formed the majority (75%). The remaining 25% of participants in both groups were not prescribed psychotropic medications. The odds ratio shows that both groups have an identical probability of falling: OR = 1 [95% CI 0.1629, 6.1387]. This indicates no association between psychoactive medications and falls for this small study cohort.

**Table 2:** Distribution of faller status and gender in medicated versus non-medicated groups Column 1 describes status according to the administration of psychoactive medications and gender breakdown within groups.

Psychoactive medication and gender	Number of participants (% of total)	Fallers (% of total)	Non-fallers (% of total)
Total number of participants	36 (100%)	28 (77.8%)	8 (22.2%)
Psychotropic administered	27 (75%)	21 (75%)	6 (75%)
Female	18	14	4
Male	9	7	2
Psychotropic not administered	9 (25%)	7 (25%)	2 (25%)
Female	7	7	0
Male	2	0	2
Female totals	25 (69.4%)	21	4
Male totals	11 (30.6%)	7	4

Twenty-one female fallers accounted for 93 (76.9%) of the falls, and seven male fallers accounted for 28 (23.1%) of the falls, giving statistical significance at p < 0.01 (Z=3.0412, p < 0.00236). Of the falls group, the majority, 24 (85.7%), were multiple fallers, with only 4 participants (14.3%) having single falls. All the fallers not prescribed psychotropic medications were female.

The non-dementia elderly residents in the same facility (n=55) had 333 falls during the study period. Including falls from the dementia group (n=121), this gives a total of 454 falls in the residential facility over the study period. These figures correspond to an overall fall rate of 6.8 falls per 1000 occupied bed-days. Falls data for both the dementia cohort and non-dementia clients are summarized in Appendix 2.

Falls and harm

All dementia study participants had a high falls risk score documented in their records, with corresponding falls prevention strategies listed in their care plans. Falls were coded according to outcome severity, with a range from 1 to 4, as follows: 1 - severe harm/death; 2 - moderate harm; 3 - mild harm; 4 - no harm/near miss. This severity rating is defined by state government policy.

One fall was allocated ISR code 1 due to a fall-related subdural hemorrhage and subsequent death; 7 were coded as 2 (moderate harm); 76 (62.8%) falls were coded as 3; the remaining 37 falls were coded as 4 (Table 3).

**Table 3:** Falls severity rating

Incident severity rating	Degree of impact	Number of falls	Harm/no harm
1	Severe harm/death	1 (0.8%)	Harm: 84 (69.4%)
2	Moderate harm	7 (5.8%)	
3	Mild harm	76 (62.8%)	
4	No harm/near miss	37 (30.6%)	No harm: 37 (30.6%)
	Total	121 (100%)	121 (100%)

Psychotropic medications

Medication charts were consulted to verify prescriptions and doses of medications administered by care staff at the time of each fall incident. The results are tabled in the appendices: Faller participants are shown in Appendix 3, and in Appendix 4 for non-faller participants. All medications were administered via the oral route in both groups. All doses administered were classified as 'low' according to prescribing guidelines.

Faller Group (n=28; ID Numbers 1-28)

A total of 121 falls occurred in this group. Participant ID numbers 1 to 21 (Appendix 4) accounted for 90 (74.4%) of these falls and were administered a psychotropic medication within 24 hours before the fall, either as a regular or PRN dose. Participant ID numbers 22 to 28 were not prescribed any psychotropic medications, and this sub-group accounted for 31 (25.6%) of the total falls in this group.

Non-Faller Group (n=8; ID Numbers 29-36)

None of these participants had a fall during the study period. As with the faller group, some of these participants were administered prescribed psychotropic medications, and some had no prescribed psychotropic medications. Appendix 3 displays the range of psychotropic medications administered to this group of participants. Participants 29-34 were administered psychotropic medications. Two participants had no prescribed psychotropic medications.

The psychotropic medication class featured the most frequently in both groups is SSRI. Clients that were administered SSRI medication had a total of 57 falls (47.1% of the total 121 falls). DSN antagonists appeared for 41 falls (33.9% - risperidone and quetiapine). SNRI medicines appeared in 12 falls (9.9%). GABA-A receptor modulators appeared in 13 falls (10.7% - temazepam, lorazepam, oxazepam). Other medications making up the total were serotonin-noradrenaline modulators (mirtazapine) and dopamine antagonists (haloperidol).

The administered medications and their corresponding NbN pharmacological domains are shown in Table 4. Falls data are presented alongside for both groups (fallers and non-fallers). Each fall is matched with medication and then grouped according to the domain.

**Table 4:** Falls and pharmacological domains implicated by pharmacotherapy SSRI: selective serotonin reuptake inhibitor; SNRI: serotonin and noradrenaline reuptake inhibitor

Pharmacological domain affected	Number of faller participants receiving pharmacotherapy	Medications concurrent with each pharmacological domain (no. of falls)	Number of falls concurrent with each pharmacological domain (% of total)	Number of non-faller participants concurrent with each pharmacological domain
Dopamine	14	Haloperidol (4)	45 (21.4%)	4 (30.8%)
		Risperidone (22)		
		Quetiapine (19)		
Serotonin	19	SSRI (57)	114 (54.3%)	3 (23.0%)
		SNRI (12)		
		Risperidone (22)		
		Quetiapine (19)		
		Mirtazapine (4)		
Noradrenaline	9	Risperidone (22)	38 (18.1%)	5 (38.5%)
		SNRI (12)		
		Mirtazapine (4)		
GABA	3	Oxazepam (3)	13 (6.2%)	1 (7.7%)
		Lorazepam (3)		
		Temazepam (7)		
		Totals	210 (100%)	13 (100%)

A total of 210 fall-domain pairs emerge from the analysis, which is more than the actual number of reported falls (n=121). This is because some clients were concurrently administered more than one psychotropic medication at a time (Appendix 3, participants 2, 3, 4, 9, 15, 17, and 19). Participant 18 has two psychotropics listed in Appendix 3, but these were prescribed and ceased at different dates and were therefore not given concurrently.

Serotonin is the pharmacological domain most represented in Table 4 (54.3% of the fall-domain total). Other domains represent the remaining falls (dopamine 21.4%, noradrenaline 18.1%, and GABA-A 6.2%). Noradrenaline and dopamine together represent almost 70% of the pharmacological domains among non-faller participants (noradrenaline 38.5% and dopamine 30.8%).

## Discussion

According to the 2020 report of the Lancet Commission [25], dementia is an increasing burden on ageing communities, families, and healthcare systems, with the worldwide number of older adults with dementia rising steadily and projected to reach 152 million people by 2050. This is thought to be related to longer life expectancies, ageing global populations, and the burden of at least 12 identified risk factors, all of them modifiable [25]. Dementia clients in aged care facilities in Australia receive care based on national guidelines, but many challenges to the effective management of elderly dementia clients remain, including agitated or aggressive behaviours and recurrent falls. This study highlights these ongoing challenges.

Most study participants (69.4%) were elderly females over the age of 80. This is in line with current observations that older women are more likely to develop major NCDs than men [25]. Several types of dementia were identified among participants, most commonly unspecified and recorded simply as 'dementia', with Alzheimer's disease being the second most frequently documented type of dementia.

Several pharmacological domains known for their psychoactive roles regarding both therapeutic effects and adverse effects were identified. These were serotonin, dopamine, noradrenaline, and gamma-amino-butyric-acid A (GABA-A). Serotonin emerged as the most frequent domain, and this was due to the usage of antidepressants such as SSRIs and antipsychotics such as risperidone.

Falls

Staff at the study facility are professional aged-care workers who promptly report all fall incidents through designated safety and quality programs. Falls incur considerable stress on residents, staff, and families, and there is a positive culture for reporting to identify areas of risk for the improvement of both care delivery and aspects of the workplace/living environment in the facility.

Fall rates in residential aged care range between 3 and 13 per 1000 OBD [28], and are generally higher than hospital fall rates, which range from 3 to 5 falls per 1000 OBD as reported in the USA [29]. The overall fall rate in this study (6.8 falls per 1000 OBD) compares with other contemporary aged care settings. A rate of 10.7 per 1000 ODB was reported in 2019 for a residential facility in Western Australia [30]. Fall rates per 1000 OBD for the dementia group in this study were not calculated as bed occupancy data are not stratified. The facility is a mixed setting with no allocated 'dementia beds'.

The majority of participants in the dementia cohort (77.8%) had at least one fall during the study period, and most of them were multiple fallers. A further observation is that most falls (74.4%) occurred on a background of psychotropic medication usage. These patterns reflect results from other dementia studies [11-13]. Most falls (69.4%) were associated with some degree of harm (Table 3).

The observed proportion of participants who had a fall was the same regardless of psychotropic medication usage, although the small sample sizes do not allow for the provision of data for testing a hypothesis.

Gender

Elderly females are known to have a higher rate of falls compared to men, although the risk factors appear to be gender-based (for example, higher household income appears to be protective against falls in women only) [24]. This phenomenon represents elderly populations in general and not dementia groups, where fewer fall studies have been conducted. This study reflects previous observations that the majority of elderly fallers are female. Females made up almost 70% of the study cohort, in line with the fact that female gender is a known risk factor for dementia [25]. The study cohort skew towards female fallers is therefore expected when these combined factors are considered.

Falls prevention and polypharmacy

Medication reviews are a crucial feature of the multi-disciplinary approach to fall prevention. This is not only because of the known increase in fall risk associated with FRIDs but also polypharmacy. FRIDs include psychotropic medications as well as other medicines such as diuretics and opioid analgesics, which are commonly prescribed for the elderly. Polypharmacy relates to the multiple prescriptions of any type of medication, not only FRIDs, and is defined as the regular and simultaneous use of five or more prescribed medications [4]. Many participants in this study belonged to this category, although data on this topic were beyond the scope of the study and were not collected.

Pharmacological mechanisms of psychoactive medications

Selected examples of commonly prescribed psychotropic medications for the management of behavioural disturbances, anxiety, and depression in adults are outlined in Appendix 1. Many of these medications were utilized in the study cohort as part of a dementia pharmacotherapy approach (Appendices 3 and 4). Four neurotransmitter domains emerge: serotonin, dopamine, noradrenaline, and GABA-A. These molecules are all amine neurotransmitters regulating central pathways of the brain responsible for the control of neurocognitive functions including executive and motor function, memory, language, and social-emotional behaviours. Serotonin domains were represented in over half of the participants in the faller group, with the other half represented in roughly equal parts by dopamine and noradrenaline. GABA-A neurotransmission was featured only marginally.

Serotonin is the most frequently represented pharmacological domain in this study. This is because the serotonin domain is targeted by several medications frequently prescribed to dementia patients, namely antidepressants and antipsychotics. Serotonin levels may be enhanced (treatment for depression) or diminished (treatment for BPSD). Both serotonin-enhancing and serotonin-blocking agents are therefore well represented in the faller group, although usually not concomitantly prescribed. However, two clients (participants 2 and 9, Appendix 3) did receive concomitant doses of an SNRI with a DSN antagonist, or doses of an SSRI with a DSN antagonist. SNRIs are not indicated for the management of depression in dementia [15] and were seen to be much less frequently prescribed than SSRIs in this study cohort.

Prescribing guidelines advise limited use of benzodiazepines and antipsychotics due to the well-documented fall risk associated with such medications. This caution is seen in the low numbers of participants taking haloperidol (n=7) and the handful of participants who were prescribed a GABA-A modulator (n=4).

Participant 19 is worth a mention (Appendix 3). This person had a total of 11 falls reported over a period of ten consecutive months. Pharmacotherapy was given in the form of quetiapine throughout this period, eventually being deprescribed after a series of recurrent falls. This participant features in Table 1 as a person with Parkinson-related dementia. It may be speculated that the increased rate of falls in people taking antipsychotics, such as this individual, could be attributed to adrenergic effects causing hypotension, which is a known fall risk factor [8], as well as other neurochemical disturbances.

Any hypothesis implicating a given pharmacological domain with an increase in fall risk is challenged by the complex neurochemistry surrounding both dementia pathology itself, medications administered for BPSD and depression, and other fall risk factors such as gender and polypharmacy. Caution is therefore warranted in the use of psychotropic pharmacotherapy in major neurocognitive disorders, especially for elderly female dementia clients.

Depression and dementia

Depression is part of the prodromal and early stages of dementia and is often treated with SSRI medications. Depressive symptoms also result from dementia, a phenomenon known as reverse causation. SSRI treatments reduce amyloid plaque formation in animal models and delay the onset of Alzheimer's diagnosis [25]. A third of the study participants (n=12) were prescribed SSRI medication, which may offer relief from depressive symptoms. However, SSRI medications are established members of the FRID group and are known to increase fall risk in older adults [3].

Study limitations

This project is a pilot study, with the inherent benefits and limitations of pilot studies. Pilot studies are instructive and provide a useful gateway for further research, highlighting improvements in quality and efficiency, testing methodologies to ensure the study design is robust, and assessing the feasibility of a larger-scale trial or study. The study site is a 34-bed facility and offers a small but adequate sample size for a pilot study (n=36). Small sample sizes may introduce bias where the chosen cohort is not representative of the broader demographic. Pilot studies do not provide for the translation of results into clinical practice or test a hypothesis, but they can provide crucial preliminary data for larger studies. Larger studies would therefore allow for the testing of hypotheses and deeper statistical analyses to ascertain the correlation between psychoactive drugs and falls in dementia. Conclusions from larger-scale studies may then provide data useful for application in clinical practice, for example, the modification of prescribing guidelines.

The study site is a general RACF and not a designated dementia unit. Aged care facilities in Australia are commonly mixed residential settings, welcoming both dementia and non-dementia clients into permanent care. From a research perspective, this presented the challenge of mixed datasets whereby it was not possible to calculate incidence rates of falls for the dementia cohort. However, individual records were accessed to allow the specific collection of data for dementia clients, which was an important focus of the study. All eligible participants were included in the study according to defined criteria, which prevented any selection bias.

Regarding confounding factors, a common limitation of any research activity, two types of confounders are described below. They relate to fall determinants and the coronavirus outbreak.

Fall Determinants

Many factors, besides psychotropic medications, contribute to falls. These factors are common across elderly age groups living in nursing homes and include antihypertensive medications [2], anticonvulsants, and opioids [4] given as treatments for other medical conditions and underlying health problems besides dementia. Non-medication-related risk factors are also identified and include other physical illnesses, cognitive decline (dementia itself is a risk factor for falls), sensory impairment and incontinence, and environmental factors such as ill-fitting footwear and cluttered living spaces [8]. This study focuses solely on psychotropic medications as a determinant of falls because published evidence suggests this subgroup of medications is indeed associated with an increased risk of falls, specifically in people with dementia [11,12]. Multifactorial confounders for falls, however, remain a limitation of this type of study.

COVID-19

With the outbreak of the Sars-Cov2 (severe acute respiratory syndrome coronavirus 2) pandemic in 2020 and the spread of COVID-19 disease, residents of nursing homes experienced frequent lockdown conditions and closure to visitors for many months. Residents, therefore, enjoyed fewer benefits from outings, activities, and social contact from friends and family. Family support and diversional therapy in the form of adapted activities are useful strategies for dementia management and contribute significantly to the prevention of falls [18]. The 2020 dementia report from the Lancet Commission discusses the impact of the current COVID pandemic on dementia clients in residential aged care [25]. It is speculated that the isolation of residents from family contact may result in the increased agitation in dementia clients who are unable to comprehend the rationales behind lockdown regimes and less able to adhere to public health advice. An increase in the use of psychotropic medications to manage agitation as well as risks associated with COVID disease for both staff and residents combine to challenge normal care routines. Staff resources were indeed stretched during the study period as the facility adapted to the pressures of COVID. Encouragingly, results from stratified falls data in this study clearly show this was not the case, as the rate of falls during COVID was one of the lowest found in the study period (Appendix 2).

Future studies

Dementia is a growing worldwide concern and a consensus on optimal evidence-based management of major NCDs has yet to be described. The findings of this research are not meaningful in terms of suggesting an association between the variables under scrutiny (due to expected study limitations as discussed above). However, some statistical outcomes were significant, and overall, this pilot study helps design larger studies investigating falls and psychotropic medications in dementia settings. Important factors that warrant continued attention in future studies are age, gender, medication subgroups and dementia subtypes (for example, Alzheimer disease as compared to Parkinson’s dementia). Special attention may be given to female dementia clients as they appear to be a high-risk group for falls. In particular, the use of NbN pharmacological domains for data analysis rather than traditional methodologies will allow a novel approach.

Future studies investigating the health and well-being of dementia clients may be facilitated through the creation of dedicated dementia units or the allocation of dementia beds within care settings. In this way, data may be collected and analysed more efficiently, avoiding the inconveniences of mixed datasets.

## Conclusions

This investigation finds that psychotropic medications continue to be prescribed to the elderly at low doses for the management of depression and behaviours related to dementia. In addition, recurrent falls were reported, mostly among females, and they were frequently associated with harm. Although the exploratory nature of the study does not test for an association between falls and psychoactive medications, the pharmacotherapy in this dementia report is shrouded in falls. The question thus emerges: 'Is psychotropic pharmacotherapy for dementia doing more harm than good?'. This pilot study serves as a basis for designing and carrying out larger future studies focusing on psychoactive medications and falls in dementia, with the hypothesis that psychoactive drugs are important risk factors for falls in people with dementia. Results of larger studies will support safe prescribing in dementia pharmacotherapy in the context of behaviour management, depression, and falls prevention.

## Appendices

Appendix 1

**Table 5:** NbN classification of selected psychotropic medications prescribed in adults 5-HT_2_: 5-hydroxytryptamine type 2 receptor; GABA-A: gamma-aminobutyric acid A; SSRI: selective serotonin reuptake inhibitor; SNRI: serotonin and noradrenaline reuptake inhibitor; D_2_: dopamine type 2 receptor.

Former classification	Medication	NbN pharmacology domain	Mechanism of action	Indication
Atypical antipsychotic	Risperidone	Dopamine, serotonin, noradrenaline	Antagonist of receptors for dopamine, serotonin, noradrenaline: Higher selectivity for 5-HT_2 _receptors relative to D_2_ receptors. Noradrenergic blockade via central α_1_ adrenergic receptor antagonism.	Agitation, aggression, or psychosis of dementia. Not indicated for dementia due to Lewy body or Parkinson’s.
	Quetiapine			
	Olanzapine	Dopamine, serotonin	Antagonist of receptors for dopamine and serotonin	Acute severe behavioural disturbance in adults.
Typical antipsychotic	Haloperidol	Dopamine	Dopamine antagonist at D_2_ receptors	Restlessness and agitation in the elderly. Violent or dangerously impulsive behaviour.
Anxiolytic	Oxazepam	Positive allosteric modulator	GABA-A receptor, benzodiazepine site	Behavioural disturbances, anxiety. If an antipsychotic or an antidepressant cannot be used, a benzodiazepine with a short half-life and no active metabolites (e.g., oxazepam) may be considered. Single dose of a benzodiazepine may be used to calm a patient to allow an investigation to be undertaken.
	Diazepam			
	Lorazepam			
Hypnotic	Midazolam			Acute severe behavioural disturbance in adults
Antidepressant	SSRI	Serotonin	Selective serotonin reuptake inhibitor	Depression and anxiety
	SNRI	Serotonin, noradrenaline	Serotonin and noradrenaline reuptake inhibitor	Depression and anxiety
	Mirtazapine	Noradrenaline, serotonin	Antagonist of noradrenaline and serotonin receptors	Major depressive disorder. Improves symptoms of depression and anxiety; promotes sleep.

Appendix 2

**Table 6:** Stratified falls and falls rates data Stratum 1 was a six-month dataset. All other strata (2-6) were 12-month datasets. The study period generated 67,113 OBDs (occupied bed-days). This figure was used to calculate falls incidence rates. The comments column of Table 4 show the occurrence of the COVID-19 outbreak as well as the bushfires in January 2020. The COVID-19 pandemic onset in 2020 led to frequent lockdowns of the facility where family members had restricted access to residents. Stratified falls data shows that the *COVID year* (stratum 6) reported one of the lowest falls rates of the study period.

	Period of data collection	Dementia clients (n=36), no. of falls	Non-dementia clients (n=55) no. of falls	Falls total for each period	Bed occupancy days	Stratified falls incidence rates (per 1000 OBD)	Comments
1	January 1–June 30, 2016	8	23	31	5823	5.3	6 month period
2	July 1, 2016 – June 30, 2017	16	64	80	17,288	6.9	
3	July 1, 2017–June 30, 2018	23	80	103	11,272	9.1	
4	July 1, 2018 – June 30, 2019	35	104	139	11,606	11.9	
5	July 1, 2019 – June 30, 2020	20	54	74	10,728	6.9	BUSHFIRE
6	July 1, 2020 – June 30, 2021	27	31	58	10,396	5.6	COVID-19
	Overall Totals	121 (26.7%)	333 (73.3%)	454 (100%)	67,113	6.8	

Appendix 3

**Table 7:** Fallers (n=28) and prescribed psychotropic medications All medications were administered via oral route. ID: participant identification number. Brackets after the medication name in indicate the number of falls events occurring at a time of administration of the medication. Where there are no brackets, only one psychotropic medication aligns with the fall(s). The comments column describes situations where psychotropic medication doses feature at some point during the study period but are not always contemporary with falls events. For example, participant 10 was deprescribed haloperidol after an initial fall in the facility but went on to experience six more falls without psychotropic usage. Participant 18, similarly, had SSRI deprescribed in 2019, but was prescribed a DSN antagonist (risperidone) in 2020 after which falls for this participant continued to be reported. DSN: D=dopamine S=serotonin N=noradrenaline; GABA-A: gamma-amino-butyric acid type A; SSRI: selective serotonin reuptake inhibitor; SNRI: serotonin and noradrenaline reuptake inhibitor; SR: slow release; PRN: 'pro re nata' - to be given as needed; BD: to be administered twice daily; mane: to be administered in the morning; nocte: to be administered in the evening.

ID	No. of falls	Psychotropic medication (no. of falls)	Prescribed dose (oral)	Prescribed frequency	Mechanism of action	Comments
1	1	Sertraline	50 mg	Daily, mane	SSRI	
2	12	Duloxetine (12)	90 mg	Daily, mane	SNRI	Frequent concurrent usage
		Risperidone (1)	0.25 mg	Regular BD	DSN antagonist	
		Risperidone (9)	0.5 mg	Regular BD		
		Risperidone	0.25 mg	Nocte PRN		
3	1	Haloperidol	0.5 mg	PRN TDS	Dopamine antagonist	No concurrent usage
		Escitalopram	20 mg	Daily, mane	SSRI	
4	3	Escitalopram (3)	5 mg	Daily, mane	SSRI	Concurrent usage for one fall only
		Haloperidol (1)	2 mg	PRN BD	Dopamine antagonist	
5	1	Mirtazapine	45 mg	Daily, nocte	SN antagonist (multimodal)	
6	2	Sertraline	50 mg	Daily, mane	SSRI	
7	2	Risperidone	0.25 mg	Daily, mane	DSN antagonist	
		Risperidone	0.25 mg	PRN nocte		
8	10	Sertraline	50 mg	Daily, mane	SSRI	
9	7	Escitalopram (1)	10 mg	Daily, mane	SSRI	Frequent concurrent usage
		Escitalopram (6)	20 mg	Daily, mane		
		Quetiapine SR (5)	200 mg	Daily, nocte	DSN antagonist and reuptake inhibitor	
		Quetiapine SR (2)	300 mg	Daily, nocte		
		Quetiapine (5)	25-50 mg	Daily, PRN		
		Temazepam (6)	10 mg	Daily, nocte	GABA-A receptor modulator	
		Temazepam (1)	10 mg	Nocte, PRN		
		Oxazepam (2)	15 mg	Daily, PRN		
		Risperidone (1)	1 mg	Regular BD	DSN antagonist	
10	7	Haloperidol (1)	0.5 mg	Daily, nocte	Dopamine antagonist	6 falls with no FRID medication
11	1	Risperidone	0.5 mg	Regular BD	DSN antagonist	
12	4	Paroxetine	20 mg	Daily, mane	SSRI	
13	8	Fluoxetine	20 mg	Daily, mane	SSRI	
14	3	Citalopram	20 mg	Daily, mane	SSRI	
15	3	Mirtazapine	15 mg	Daily, nocte	SN antagonist (multimodal)	Frequent concurrent usage
		Risperidone	0.25 mg	Daily, BD	DSN antagonist	
16	2	Quetiapine	25 mg	Daily, nocte	DSN antagonist and reuptake inhibitor	
		Quetiapine	7.5 mg	PRN nocte		
17	1	Oxazepam	7.5 mg	Daily, mane	GABA-A receptor modulator	
		Escitalopram	10 mg	Daily, mane	SSRI	
		Haloperidol	0.5 mg	Daily, mane	Dopamine antagonist	
18	7	Risperidone (1)	0.5 mg	Daily, nocte	DSN antagonist	No concurrent usage. 5 falls with no FRID medication.
		Fluoxetine (1)	20 mg	Daily, mane	SSRI	
19	11	Quetiapine (1)	100 mg	Regular nocte	DSN antagonist and reuptake inhibitor	Regular quetiapine 300 mg was ceased in 2020 after 11 falls from 2019 to 2020
		Quetiapine (10)	300 mg	Regular nocte		
		Quetiapine (1)	50 mg	PRN nocte		
		Quetiapine (1)	100 mg	PRN nocte		
		Quetiapine (2)	200 mg	PRN nocte		
		Lorazepam (3)	0.5 mg	Regular nocte	GABA-A receptor modulator	
		Lorazepam (1)	0.5 mg	PRN nocte		
20	2	Risperidone	0.5 mg	Regular BD	DSN antagonist	
21	2	Risperidone	0.5 mg	Regular BD	DSN antagonist	
22	4	NONE				These participants were not prescribed any psychotropic medications
23	2	NONE				
24	9	NONE				
25	8	NONE				
26	3	NONE				
27	3	NONE				
28	2	NONE				
Total	121					

Appendix 4

**Table 8:** Non-fallers (n=8) and prescribed psychotropic medications All medications were administered via oral route. ID: participant identification number; DSN: D=dopamine S=serotonin N=noradrenaline; GABA-A: gamma-amino-butyric acid type A; SSRI: selective serotonin reuptake inhibitor; SNRI: serotonin and noradrenaline reuptake inhibitor; SR: slow release; PRN: 'pro re nata' - to be given as needed; BD: to be administered twice daily; mane: to be administered in the morning; nocte: to be administered in the evening.

ID	Psychotropic medication	Dose	Frequency	Mechanism of action
29	Risperidone	4 mg	Daily, nocte	DSN antagonist
30	Risperidone	0.25 mg	Regular BD	DSN antagonist
	Temazepam	10 mg	PRN nocte	GABA-A receptor modulator
31	Risperidone	0.25 mg	BD	DSN antagonist
	Haloperidol	0.5 mg	PRN hourly	Dopamine antagonist
32	Venlafaxine SR	150 mg	Daily, mane	SNRI
	Haloperidol	0.5 mg	PRN	Dopamine antagonist
33	Venlafaxine SR	150 mg	Daily, mane	SNRI
34	Escitalopram	10 mg	Daily, mane	SSRI
35	NONE			These participants were not prescribed any psychotropic medications
